# Leniolisib: a novel treatment for activated phosphoinositide-3 kinase delta syndrome

**DOI:** 10.3389/fphar.2024.1337436

**Published:** 2024-02-12

**Authors:** Surya K. De

**Affiliations:** ^1^ Conju-Probe, San Diego, CA, United States; ^2^ Bharath University, Department of Chemistry, Chennai, Tamil Nadu, India

**Keywords:** idelalisib, duvelisib, copanlisib, alpelisib, leniolisib, phosphatidylinositol 3-kinase

## Abstract

**Figure:**
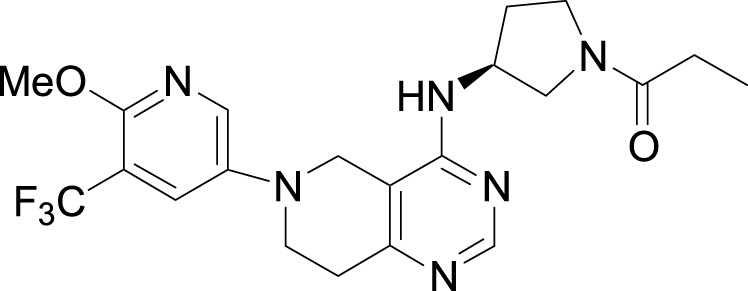
IC_50_ = 11 nM (PI3Kδ); 244 nM (PI3Kα); 424 nM (PI3Kβ), 2,230 nM (PI3Kγ).

IC_50_ = 11 nM (PI3Kδ); 244 nM (PI3Kα); 424 nM (PI3Kβ), 2,230 nM (PI3Kγ).

## 1 Introduction

The PI3K/AKT/mTOR network is a crucial cellular signaling pathway that controls several biological processes including cell growth and proliferation, cell survival, protein synthesis, and glycolysis metabolism (Barile et al., 2010; Martini et al., 2014; Hoegenauer et al., 2016; Hoegenauer et al., 2017; Rao et al., 2017; Avery et al., 2018; Cannons et al., 2018; Michalovich and Nejentsev, 2018; Jamee et al., 2020; Sogkas et al., 2020; Bloomfield et al., 2021; Wang et al., 2022; Bou Zeid and Yazbeck, 2023; Cant et al., 2023; Nguyen et al., 2023; Rao et al., 2023). Phosphatidylinositol 3-kinase is a family of lipid kinases consisting of an enzymatic p110δ subunit and a regulatory p85 subunit that is expressed predominantly in hematopoietic cells. PI3K converts phosphatidylinositol-4,5-bisphosphate (PIP2) to phosphatidylinositol-3,4,5-trisphosphate (PIP3), a signaling molecule whose mutation results in cancer. Activated phosphoinositide 3-kinase delta (PI3Kδ) syndrome (APDS 1) is an inborn error of immunity, caused by mutations in catalytic p110δ (*PIK3CD*), whereas *pIK3CD* variants are activating/gaining function in *PIK3R1* encoding the regulatory subunit p85α, which causes APDS2 (Rao et al., 2017; Bloomfield et al., 2021).

The approved PI3K kinase inhibitors are idelalisib, duvelisib, copanlisib, alpelisib, and umbralisib (Hoegenauer et al., 2016; Rao et al., 2017; Bloomfield et al., 2021; Bou Zeid and Yazbeck, 2023). Idelalisib, a purine-quinazolin-4-one derivative was approved in 2014 for the treatment of patients with relapsed chronic lymphocytic leukemia (CLL). It is a first-in-class PI3K-δ selective inhibitor. It also inhibits AKT, MAPK, TNFα, CXCR4, and CXCR5 in cell-based assays. Replacing one *N*-atom with carbon in the quinazoline ring and changing F to Cl yielded duvelisib, which is a selective PI3K-γ and PI3K-δ inhibitor. Duvelisib was approved in 2018 for the treatment of adult patients with relapsed or refractory CLL or SLL and relapsed or refractory FL after at least two prior therapies. Duvelisib also inhibits several cell-signaling pathways such as B-cell receptor signaling, CXCR12-mediated chemotaxis of malignant B cells, and CXCL12-induced T cell migration. Copanlisib, a-2,3-dihydroimidazo[1,2-c]quinazolin-5-yl-pyrimidine derivative was approved in 2017 as a PI3K-α and PI3K-δ inhibitor for the treatment of adult patients with relapsed follicular lymphoma (FL) who have received at least two prior systemic therapies. Alpelisib, a small molecule was approved in combination with fulvestrant for the treatment of postmenopausal women, and men, with hormone receptor (HR)-positive, human epidermal growth factor receptor 2 (HER2)-negative, PIK3CA-mutated, advanced, or metastatic breast cancer as determined by an FDA-approved test. It is a selective PI3Kα inhibitor. Umbralisib, a 4 *H*-chromen-4-one derivative was approved in 2021 for the treatment of adult patients with relapsed or refractory marginal zone lymphoma (MZL) who have received at least one prior anti-CD20-based regimen. Umbralisib selectively inhibits PI3K-delta and casein kinase 1-epsilon. Most PI3K inhibitors have been approved based on single-arm studies but were later withdrawn due to severe adverse effects. There is an urgent need for a new PI3K inhibitor with a good safety profile. Leniolisib was approved on 26 March 2023, based on a 12-week blinded, randomized, placebo-controlled study in adult and pediatric patients 12 years of age and older with an APDS-associated PI3Kδ genetic mutation [NCT02435173]. Chemical structure, PI3K isoform selectivity, primary disease indications, and serious adverse events of approved PI3K inhibitors are summarized in Table 1.

**TABLE 1:** Chemical structure, biological data, disease indications, and adverse events of Approved PI3K inhibitors.

Chemical structure	PI3K	Primary disease indications	Serious
	Isoform specificity IC_50_ (nM)		Adverse events (Grade ≥3)
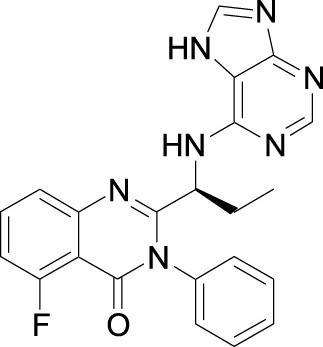 Idelalisib	PI3Kδ: 2.5	CLL, SLL, iNHL	Pneumonia, sepsis, Diarrhea/colitis, urinary tract infection, abdominal pain, Cutaneous reactions, Hepatotoxicity
	PI3Kγ: 89		
	PI3Kα: 8600		
	PI3Kβ: 4000		
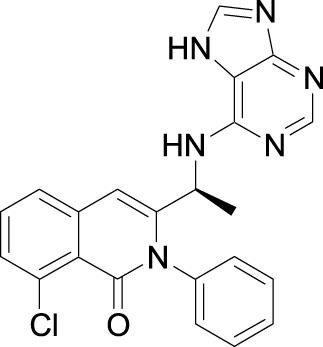 Duvelisib	PI3Kδ: 2.5	CLL, SLL, FL	Pneumonia, Diarrhea/colitis, Abdominal pain, Hepatotoxicity, Neutropenia, Anemia, Hepatotoxicity, Fatigue
	PI3Kγ: 27		
	PI3Kα: 1602		
	PI3Kβ: 85		
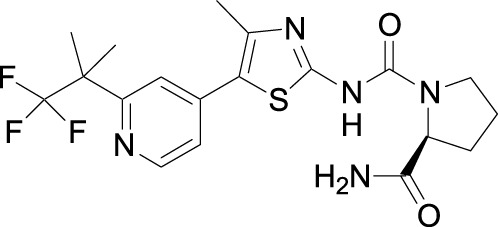 Alpelisib	PI3Kα: 4.6	Breast cancer	Pneumonia, Cutaneous reactions, Diarrhea/colitis, Hyperglycemia, Abdominal pain, Fatigue
	PI3Kγ: 250		
	PI3Kδ: 290		
	PI3Kβ: 1200		
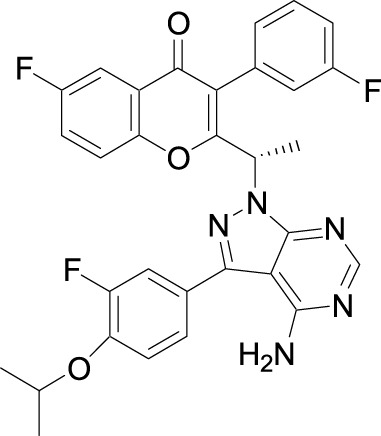 Umbralisib	PI3Kδ: 22	FL, ML	Pneumonia, Diarrhea/colitis, Hepatotoxicity, Cutaneous reactions
	PI3Kγ: 330		
	PI3Kβ: 660		
	PI3Kα: 22,000		
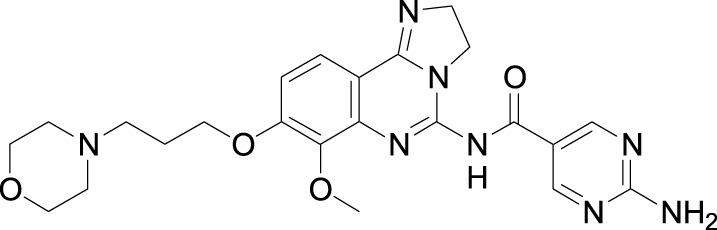 Copanlisib	PI3Kα: 0.5	FL, iNHL	Pneumonia, Hyperglycemia, Diarrhea/colitis, Hepatotoxicity, Hyperglycemia, Hypertension, Leukopenia, Neutropenia
	PI3Kβ: 3.7		
	PI3Kδ: 0.7		
	PI3Kγ: 6.4		
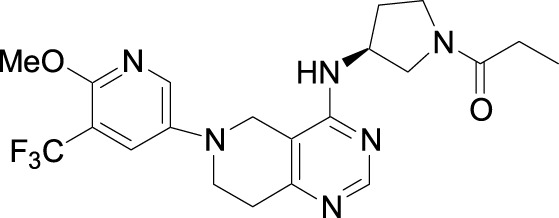 Leniolisib	PI3Kδ: 0.01	APDS	No Grade 3 Adverse events
	PI3Kα: 0.24		
	PI3Kβ: 0.42		
	PI3Kγ: 2.23		

## 2 Physicochemical properties of leniolisib

Brand name: Orserdu; Chemical name: 1-[(3*S*)-3- [[5,6,7,8-Tetrahydro-6-[6-methoxy-5-(trifluoromethyl)-3-pyridinyl]pyrido[4,3-*d*]pyrimidin4-yl]amino]-1-pyrrolidinyl]-1-propanone phosphate (1:1); Chemical formula: C_21_H_25_F_3_N_6_O_2_•H_3_PO_4_; Molecular weight: 450.47 for the free base, 548.46 for the phosphate salt; Topological Polar Surface Area: 83.5 Å^2^; Hydrogen Bond Donor Count: 1; Hydrogen Bond Acceptor Count: 8; Rotatable Bond Count: 5; Heavy Atom Count: 32; Number of Ring Count: 4; LogD (pH 7.4): 3.1; Solubility: the solubility of leniolisib phosphate is pH dependent with decreasing solubility with increasing pH; Rule of 5 Violations: 0 (Hoegenauer et al., 2016; Hoegenauer et al., 2017).

## 3 Development of leniolisib

**FIGURE 1:**
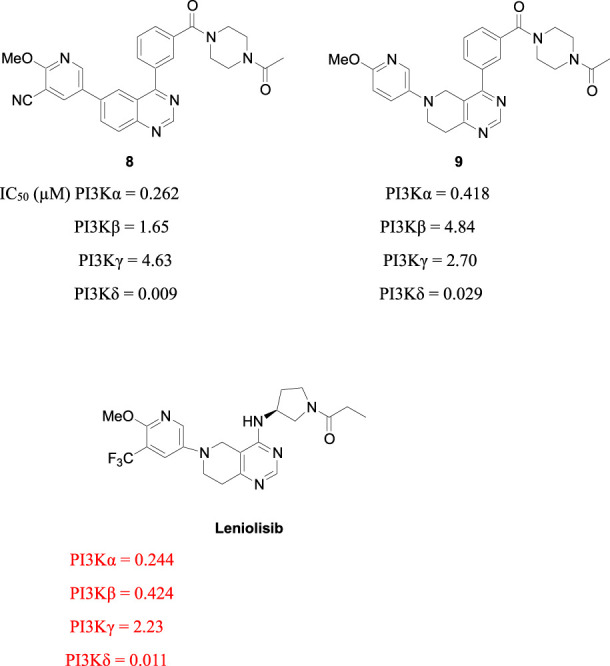
Optimization of quinazoline derivatives.

Investigators from Novartis discovered novel 4,6-diaryl quinazolines (Hoegenauer et al., 2016; Hoegenauer et al., 2017) such as compound **8** (Figure 1). It is a potent and isoform-selective PI3Kδ inhibitor *in vitro* and *in vivo*. However, the poor water solubility of this compound leads to an unfavorable PK profile in rats (Hoegenauer et al., 2016). They modified the core structure to partially saturated bicyclic systems resulting in compound **9**. Compound **9** is also an isoform-selective potent PI3δ inhibitor but the cellular potency decreased 54 times compared to compound **8** due to low cellular permeability. Researchers introduced a spacer moiety such as ether or NH at the 4-position of the core structure, improving solubility, membrane permeability, and biochemical activity in cellular assays. By introducing a CF3 group at the 3-position of the methoxypyridine ring and at the 4-position with pyrrolidinyl-1-propanone with an NH spacer, a clinical candidate, leniolisib, was obtained. Lenilisib shows an optimal profile, good solubility (500 times better than the initial compound), metabolic stability, membrane permeability, and favorable PK properties (Hoegenauer et al., 2017).

## 4 Synthesis

**SCHEME 1:**
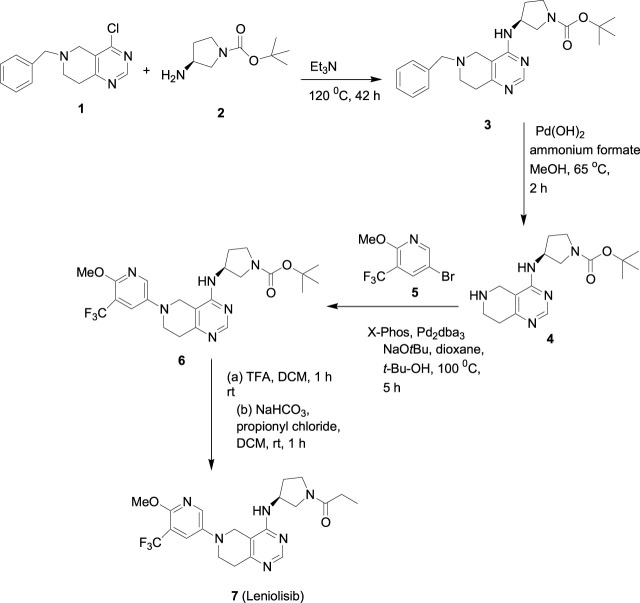
Synthesis of leniolisib.

The synthesis of leniolisib starts with compound **1** as shown in Scheme 1 (Hoegenauer et al., 2016; Hoegenauer et al., 2017).

6-Benzyl-4-chloro-5,6,7,8-tetrahydro-pyrido[4,3-*d*]pyrimidine (compound **1**) is coupled with (*S*)-*tert*-butyl 3-aminopyrrolidine-1-carboxylate (compound **2**) in the presence of triethylamine at 120 °C for 42 h to give compound **3** a 93% yield. The benzyl group is deprotected with 20% palladium hydroxide on carbon and ammonium formate in methanol at 65 °C for 2 h to give compound **4** a 66% yield. Compound **4** is coupled with 5-bromo-2-methoxy-3-(trifluoromethyl)pyridine (compound **5**) in the presence of sodium -*tert*-butoxide, tris(dibenzylideneacetone)dipalladium(0), 2-di-*t*-butylphosphino-2'-(*N*,*N*-dimethylamino)biphenyl in t*ert*-butanol at 100 °C for 5 h to give compound **6** a 74% yield. Deprotection of the Boc group in DCM/TFA, followed by coupling with propionyl chloride in the presence of sodium bicarbonate in DCM at room temperature for 1 h gives the final compound **7** (leniolisib) a 76% yield.

## 5 Dosage and administration

The recommended dosage is 70 mg orally twice daily approximately 12 h apart, with or without food in adult and pediatric patients 12 years of age and older and weighing ≥45 kg.

## 6 Mechanism of action of leniolisib

APDS is linked with gain-of-function variants in the gene encoding p110δ *PIK3CD* or loss of function variants in the gene encoding p85α *PIK3R1*, each of which causes hyperactivity of PI3K-delta (Fresno Vara et al., 2004; Hoegenauer et al., 2016; Hoegenauer et al., 2017; Rao et al., 2017; De Buck et al., 2018; Michalovich and Nejentsev, 2018; Pearson et al., 2019; Bou Zeid and Yazbeck, 2023; Cant et al., 2023; Rao et al., 2023). PI3Kδ homeostasis is an important step in the development and function of both B and T cells. In cell-free isolated enzyme assays, leniolisib selectively inhibits PI3K-delta (IC_50_ = 11 nM) over PI3K-alpha (22-fold), PI3K-beta (38-fold), PI3K-gamma (202-fold), and other kinases. In cell-based assays, leniolisib inhibits pAKT pathway activity resulting in inhibition of proliferation and activation of B and T cell subsets (Brown et al., 2022).

The primary endpoint of leniolisib is safety and tolerability in patients with APDS. Leniolisib met the primary endpoint with all adverse events being mild and grades 1–3 (Rao et al., 2023).

The secondary endpoint of leniolisib is the efficacy in APDS patients. From the clinical trial, leniolisib for the treatment of patients with APDS showed rapid normalization of the PI3Kδ signaling pathway, reduction of lymphoproliferation, and improvement of key immune cell subsets (Cant et al., 2023; Rao et al., 2023). A significant reduction in lymphadenopathy was observed in patients treated with leniolisib (62.7% reduction in lymph node size and 37.6% reduction in spleen volume) compared to placebo (5%).

## 7 Pharmacodynamics

Leniolisib reduced PI3Kδ pathway hyperactivation in cell lines overexpressing p110δ mutants and primary cells. Within the recommended dose ranges, higher leniolisib plasma concentrations were associated with greater reductions in pAkt-positive B cells. Treatment with leniolisib 70 mg twice daily doses at steady state was estimated to produce a time-averaged reduction in pAkt-positive B cells of almost 80% (Hoegenauer et al., 2016; Hoegenauer et al., 2017; Rao et al., 2017; Bou Zeid and Yazbeck, 2023).

## 8 Pharmacokinetics (PK)

Steady-state drug concentrations were reached after approximately 2–3 days of treatment. The pharmacokinetics of leniolisib are similar in both healthy participants and APDS patients (De Buck et al., 2018; Pearson et al., 2019).

### 8.1 Absorption

The systemic drug exposure (AUC and C_max_) of leniolisib increases in a dose-dependent manner.

The median time to maximum plasma concentration (T_max_) of leniolisib is 1 h and is independent of dose. Food has no significant effect.

### 8.2 Distribution

The volume distribution of leniolisib is almost 28.5 L in patients with APDS and 94.5% of it binds to human plasma proteins.

### 8.3 Elimination

The terminal elimination half-life of leniolisib is 10 h and the apparent oral clearance is 4 L/h (De Buck et al., 2018; Pearson et al., 2019).

### 8.4 Metabolism

**SCHEME 2:**
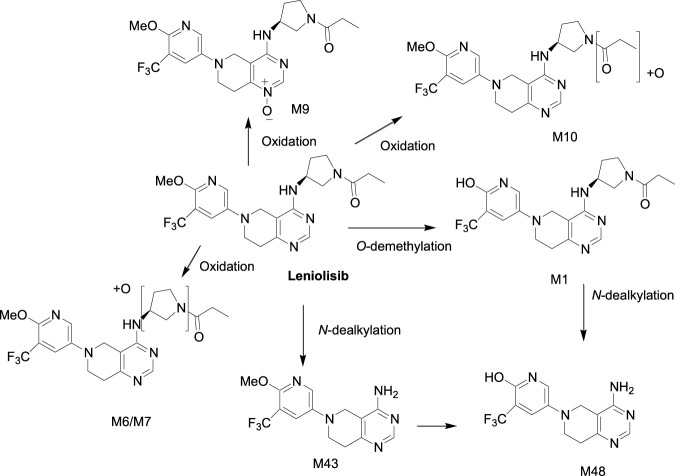
Metabolism of leniolisib.

Leniolisib is primarily metabolized in the liver by CYP3A4 (94.5%) in the oxidative metabolism pathway with minor contributions from other enzymes (3.5% CYP3A5, 0.7% CYP1A2 and 0.4% CYP2D6). The main circulating species is the parent drug (Fresno Vara et al., 2004; Hoegenauer et al., 2016; Hoegenauer et al., 2017; Cant et al., 2023). It undergoes *O*-demethylation to form the M1 metabolite as shown in Scheme 2. The *N*-dealkylation gives the M43 metabolite. Oxidation of the *N*-atom on the pyrimidine forms the M9 metabolite. Several oxidations of leniolisib yield M6/M7 and M10 metabolites, which have not been fully characterized (De Buck et al., 2018; Pearson et al., 2019).

### 8.5 Excretion

Leniolisib is excreted in the feces (67%) and urine (25.5%).

## 9 Drug interaction studies

### 9.1 Effect of other drugs on leniolisib

Leniolisib is a substrate of CYP3A4. Concomitant use of leniolisib with any strong CYP3A4 inhibitors (NCT02435173) should be avoided.

### 9.2 Effect of leniolisib on other drugs

Leniolisib is an inhibitor of CYP1A2. Concomitant use of leniolisib with any strong CYP1A2 inhibitors should be avoided. Leniolisib is an inhibitor of BCRP, OATP1B1, and OATP1B3. Concomitant use of leniolisib with these inhibitors may reduce the efficacy of leniolisib (NCT02435173).

## 10 Adverse reactions

The most common adverse reactions (>10%) observed during clinical trials were headache, sinusitis, atopic dermatitis, tachycardia, diarrhea, fatigue, pyrexia, back pain, neck pain, and alopecia (NCT02435173).

## 11 Conclusion

The first generation of clinical PI3Kδ and/or PI3Kγδ-selective inhibitors such as idelalisib (PI3Kδ), duvelisib (PI3Kγδ), and umbralisib (PI3Kδ) have demonstrated antitumor activity in R/R iNHL, CLL, and FL from single-arm clinical trials. Initially, these agents showed favorable results in terms of overall response rate (ORR) and progression-free survival (PFS). However, in double-blind randomized clinical trials, these agents showed a decrease in overall survival (OS) and an increase in fatal and severe adverse reactions compared with patients in the control arms. Leniolisib has demonstrated a good safety profile due to its significant chemical and structural differences from the previous PI3K inhibitors. It is approved for the treatment of activated phosphoinositide 3-kinase delta (PI3Kδ) syndrome. Previously, Activated Phosphoinositide-3 Kinase Delta Syndrome was treated based on anti-infective prophylaxis, including antibiotics, immunoglobulin replacement, and immunomodulatory agents, such as sirolimus but inborn errors of immunity cannot be prevented by antibiotic/antiviral therapy - only hematopoietic stem cell transplantation (HSCT). HSCT therapy can improve some clinical symptoms of APDS, but patients are at high risk of engraftment failure and need unplanned donor cell infusions. This method is associated with adverse events such as graft-versus-host disease, organ toxicity, severe infectious complications, and death (Coulter et al., 2017; Maccari et al., 2018; Durandy and Kracker, 2020).

Leniolisib is the first approved drug for this disease that directly targets a cell signaling pathway. GSK has developed a new clinical candidate, Nemiralisib (**GSK2269557**) as a competitor of lenilisib and has entered it into clinical trials. However, this drug for APDS 1 has now been withdrawn due to toxicity. Nemiralisib is in clinical trials for Chronic Obstructive Pulmonary Disease (COPD) and asthma. Leniolisib is the only standalone approved drug for patients with APDS1.

Currently, leniolisib is also undergoing a clinical trial in patients with primary Sjögren’s Syndrome. Sjögren’s syndrome is a chronic (long-lasting) autoimmune disorder. It occurs when the immune system damages the glands that produce and control moisture in the eyes, mouth, and other parts of the body. The main symptoms are dry eyes and mouths. Sjögren’s syndrome is associated with high PI3K*δ* activity (Yin et al., 2022). Leniolisib alone or in combination with other drugs may be beneficial in patients with autoimmune diseases such as rheumatoid arthritis, Sjögren’s syndrome, and systemic lupus erythematosus where PI3Kδ is overactive.

## Data Availability

The raw data supporting the conclusion of this article will be made available by the authors, without undue reservation.

## Abbreviations

APDS, Activated Phosphoinositide 3-kinase Delta Syndrome; BCRP, Breast Cancer Resistance Protein; OATP1B1, Organic Anion Transporting Polypeptide 1B1; CLL, Chronic Lymphocytic Leukemia; SLL, Small Lymphocytic Lymphoma; CXCR4, C-X-C Motif Chemokine Receptor 4 Small Lymphocytic Lymphoma; iNHL, indolent non-Hodgkin Lymphomas; FL, Follicular Lymphoma; ML, marginal-zone Lymphoma; ORR, overall response rates; OS, Overall survival; PFS, Progression-free survival; PI3K, phosphoinositide 3-kinase; mTOR, mammalian target of rapamycin; PIP2, phosphatidylinositol 4,5-bisphosphate; PIP3, phosphatidylinositol 3,4,5-triphosphate; R/R, relapsed or refractory; TNF-α, Tumor necrosis factor alpha; MAPK, Mitogen-activated protein kinase.
